# Extra Virgin Olive Oil (EVOO) Improves Vascular Endothelial Function and Hemodynamic Parameters in Patients with Hyperlipidemia: A Post Hoc Analysis of a Randomized Controlled Trial

**DOI:** 10.3390/nu17233650

**Published:** 2025-11-21

**Authors:** Christos Kourek, Emmanouil Makaris, Vassiliki Benetou, Prokopios Magiatis, Virginia Zouganeli, Stavros Dimopoulos, Georgios Georgiopoulos, Alexandros Briasoulis, Ioannis Paraskevaidis, Eleni Melliou, Philippos Orfanos

**Affiliations:** 1 Department of Cardiology, 417 Army Share Fund Hospital of Athens (NIMTS), 11521 Athens, Greece; 2 Department of Hygiene, Epidemiology and Medical Statistics, School of Medicine, National and Kapodistrian University of Athens, 11527 Athens, Greece; vbenetou@med.uoa.gr (V.B.); phorfanos@med.uoa.gr (P.O.); 3 Cardiology Department, General Hospital of Messinia, 24100 Kalamata, Greece; manolismakaris@gmail.com; 4 Laboratory of Pharmacognosy & Chemistry of Natural Products, Department of Pharmacy, National and Kapodistrian University of Athens, University Campus, 15771 Zografou, Greece; magiatis@pharm.uoa.gr (P.M.); emelliou@pharm.uoa.gr (E.M.); 5 Department of Cardiology, Athens University Hospital Attikon, Medical School, National and Kapodistrian University of Athens, 12462 Athens, Greece; virginia_noa@yahoo.gr (V.Z.); georgiopoulosgeorgios@gmail.com (G.G.); 6 Cardiac Surgery Intensive Care Unit, Onassis Cardiac Surgery Center, 17674 Kallithea, Greece; stdimop@gmail.com; 7 Department of Clinical Therapeutics, School of Medicine, National and Kapodistrian University of Athens, 11528 Athens, Greece; alexbriasoulis@gmail.com; 8 School of Medicine, National and Kapodistrian University of Athens, 11527 Athens, Greece; iparas@otenet.gr

**Keywords:** extra virgin olive oil (EVOO), hyperlipidemia, endothelial function, near infrared spectroscopy (NIRS), arterial blood pressure, inflammation

## Abstract

**Background/Objectives**: Extra virgin olive oil (EVOO) exhibits potent antioxidant and anti-inflammatory properties. Recent findings from our Institute indicate that its bioactive compounds enhance lipid metabolism, suggesting EVOO as a promising nutritional intervention for hyperlipidemic patients. This clinical study aimed to evaluate the effects of EVOO consumption on vascular endothelial function and hemodynamic parameters in hyperlipidemic individuals, while comparing two EVOO types differing in polyphenol content and dosage. **Methods**: This post hoc analysis included 70 participants: 50 patients with hyperlipidemia and 20 healthy controls. All participants consumed EVOO daily for four weeks. Hyperlipidemic patients were randomized into two subgroups: one receiving high-phenolic EVOO at a low dose and another receiving low-phenolic EVOO at a high dose, ensuring equivalent total polyphenol intake. Vascular endothelial function, assessed via near-infrared spectroscopy (NIRS), served as the primary endpoint, while arterial blood pressure and heart rate were secondary endpoints. Statistical analyses employed mixed linear models. **Results**: Hyperlipidemic patients exhibited significant improvements in endothelial function, with increased reperfusion rate (*p* = 0.010) and oxygen consumption rate (*p* < 0.001) compared to controls. Reductions in maximum hyperemia time (*p* = 0.004) and hyperemia recovery time (*p* < 0.05) further indicated enhanced vascular function. Diastolic blood pressure (*p* = 0.007) and heart rate (*p* = 0.004) decreased significantly. Among subgroups, high-phenolic EVOO at lower doses was more effective in reducing systolic blood pressure (*p* = 0.049) and improving reperfusion rate (*p* = 0.049). **Conclusions**: EVOO consumption improved endothelial function and hemodynamic parameters in hyperlipidemic patients, with high-phenolic EVOO demonstrating superior vascular benefits at lower doses.

## 1. Introduction

Extra-virgin olive oil (EVOO), derived from the fruit of *Olea europaea* L., consists predominantly of monounsaturated fatty acids (MUFA) (70–80% oleic acid) and a diverse array of minor bioactive constituents. Its phenolic fraction includes hydroxytyrosol and tyrosol derivatives, oleocanthal, oleacein, and a range of secoiridoids [1,2], which collectively contribute to its antioxidant, anti-inflammatory, and lipid-modulating properties [3,4,5]. During the last decade, many studies have reported reductions in systemic inflammatory markers and oxidative stress with EVOO or phenolic-rich preparations [6], alongside improvements in HDL functionality [7,8,9] and attenuation of LDL oxidation [10,11,12]. These effects are linked to NF-κB downregulation [13], NLRP3 inflammasome restraint [14], and antioxidant activity of phenols [15]. Moreover, olive oil polyphenols help in protection of LDL particles from oxidative damage, a proximate step in atherogenesis [10,11]. A recent trial from our Institute [16] showed that daily EVOO consumption significantly improved the lipid profile of hyperlipidemic patients by increasing HDL cholesterol and reducing lipoprotein(a), indicating that high-phenolic EVOO is beneficial for cardiometabolic risk profiles when is incorporated into diet patterns.

Extra virgin olive oil (EVOO) consists predominantly of monounsaturated fatty acids (70–80% oleic acid) and a diverse array of minor bioactive constituents. Its phenolic fraction includes hydroxytyrosol and tyrosol derivatives, oleocanthal, oleacein, and a range of secoiridoids, which collectively contribute to its antioxidant, anti-inflammatory, and lipid-modulating properties. Several of these compounds, particularly hydroxytyrosol, oleuropein aglycone, and oleocanthal, have been implicated in the regulation of lipid metabolism through mechanisms such as reduced LDL oxidation, enhancement of HDL functionality, inhibition of inflammatory pathways (NF-κB, COX enzymes), and improvement of endothelial nitric oxide availability. Clinically, EVOO consumption has been associated with modest reductions in LDL cholesterol, improved LDL particle oxidation resistance, and favorable changes in overall lipid profile, supporting its role as a cardioprotective dietary component.

Vascular endothelial function is a key factor in maintaining cardiovascular health. The endothelium regulates vascular tone, anti-inflammatory response, and hemostasis. Dysfunction of the vascular endothelium is considered an early marker of cardiovascular disease [17,18]. In hyperlipidemic conditions, excess apoB-lipoproteins infiltrate the intima and undergo oxidative modification, provoking endothelial inflammation, reduced nitric-oxide (NO) bioavailability, and impaired vasodilation [19,20,21,22]. Oxidized LDL activates endothelial adhesion molecules and ROS production, promotes eNOS “uncoupling” that shifts the enzyme from NO to superoxide generation, and amplifies leukocyte recruitment and vasoconstrictor tone [22,23]. All these processes degrade endothelial barrier function and favor atherogenesis. Contemporary reviews highlight the central roles of oxidative stress, inflammation-driven signaling, and eNOS dysregulation as interconnected drivers and therapeutic targets of endothelial dysfunction in hyperlipidemia [24].

**Figure 1:**
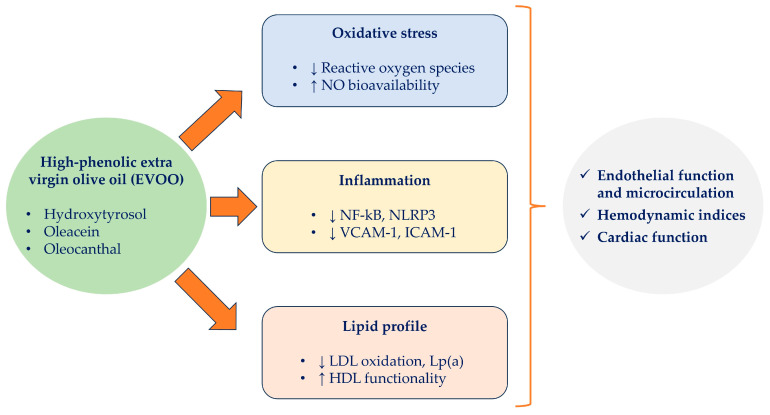
Proposed mechanisms linking high-phenolic extra virgin olive oil (EVOO) to improved endothelial and microvascular function. Phenolic constituents of EVOO (hydroxytyrosol, oleacein, oleocanthal) modulate three major pathways: (1) reduction in oxidative stress with decreased reactive oxygen species (ROS) and increased nitric oxide (NO) bioavailability; (2) attenuation of inflammatory signaling, including downregulation of NF-κB, NLRP3, and endothelial adhesion molecules (VCAM-1, ICAM-1); and (3) improvement of lipoprotein profile, with decreased LDL oxidation and lipoprotein(a) and enhanced HDL functionality. These effects converge to improve endothelial function, hemodynamic indices and cardiac function. ↑, increase; ↓, decrease.

Among the numerous phenolic constituents of EVOO, several have been mechanistically linked to modulation of endothelial biology. Hydroxytyrosol and oleacein exert strong antioxidant activity by directly scavenging reactive oxygen species (ROS) and preserving NO bioavailability, thereby improving endothelium-dependent vasodilation [25]. Oleocanthal exhibits anti-inflammatory properties through inhibition of cyclooxygenase enzymes and attenuation of NF-κB-mediated endothelial activation [26], while both hydroxytyrosol and oleuropein aglycone have been shown to downregulate adhesion molecules such as VCAM-1 and ICAM-1 [27,28]. These molecular effects correlate with reduced LDL oxidation, improved HDL-mediated cholesterol efflux, and decreased leukocyte recruitment, processes central to maintenance of microvascular integrity [22]. Collectively, these findings indicate that specific EVOO phenolics are not only bioactive but directly linked to vascular functional improvements, supporting their relevance as key drivers of the endothelial benefits observed in nutritional interventions (Figure 1).

The main hypothesis was that the consumption of EVOO with a high content of polyphenols has a beneficial effect on vascular endothelial function in patients with hyperlipidemia. The purpose of the present clinical study was to evaluate the effect of EVOO on markers of vascular endothelial function and hemodynamic parameters in patients with hyperlipidemia, as well as to compare EVOOs of different concentrations of polyphenols but with the same total polyphenol intake.

To our knowledge, this study is among the first to evaluate the microvascular and endothelial effects of high-phenolic extra virgin olive oil using near-infrared spectroscopy (NIRS) combined with the vascular occlusion technique (VOT). While previous trials have primarily relied on conduit-artery flow-mediated dilation (FMD) or biochemical markers, the integration of NIRS–VOT provides a more dynamic and sensitive assessment of microcirculatory responsiveness. This methodological innovation represents a novel approach in the field of olive oil research and enables the exploration of early microvascular adaptations to phenolic-rich dietary interventions in hyperlipidemic patients.

LDL, low density lipoprotein; HDL, high density lipoprotein; Lpa, lipoprotein a; VCAM-1, vascular cell adhesion molecule 1; ICAM-1, intercellular adhesion molecule 1; NLRP3, NLR family pyrin domain containing 3; NF-kB, nuclear factor kappa-light-chain-enhancer of activated B cells.

## 2. Materials and Methods

### 2.1. Study Design and Participants

This study is a post hoc analysis of a single blind randomized clinical trial, conducted at the General Hospital of Messinia in Greece between October 2021 and March 2022 [16]. The protocol received approval from the Scientific Council of the General Hospital of Messinia (23/29.12.2020) and was conducted in accordance with the ethical principles of the Declaration of Helsinki. Informed consent was obtained from each participant.

Diagnosis of hyperlipidemia in outpatients attending the Lipid Clinic of the Cardiology Department was based on medical history, clinical evaluation, and laboratory tests. Sampling, as well as inclusion and exclusion criteria, have been previously described in detail [16].

Participants meeting the protocol’s criteria were invited during routine follow-up. Fifty patients were assigned to two intervention groups using stratified randomization by age (≤50 or >50 years) and LDL cholesterol level (≤150 mg/dL or >150 mg/dL/cardiovascular risk), with balanced allocation within four strata. An additional 20 age- and gender-matched healthy individuals without hyperlipidemia formed a comparison group. Age was dichotomized at 50 years because endothelial function and lipid metabolism undergo clinically meaningful shifts around midlife, and previous cardiovascular studies have used this threshold to distinguish lower- from higher-risk populations [29]. Given the relatively modest sample size, using multiple age intervals would have resulted in small and unbalanced subgroups, reducing the effectiveness of stratified randomization. The ≤50 and >50-year categories therefore offered an optimal balance between clinical relevance and statistical stability.

### 2.2. Intervention

The clinical trial compared two EVOOs with different phenolic concentrations at different dosages; the lower-phenolic EVOO with 414 mg/kg at higher dosages of 20 g daily, and the higher-phenolic EVOO with 1021 mg/kg at lower dosages of 8 g daily. The aim was to keep the daily polyphenol intake constant in both groups. Participants consumed EVOO in their original form each morning while fasting for faster absorption of the phenols and were instructed to maintain their usual dietary habits throughout the study and were not placed on any restrictive dietary regimen. The only requirement was to avoid additional polyphenol-rich foods, such as olives, other olive oils, berries, red wine, dark chocolate, nuts, green tea, and polyphenol-containing supplements, to prevent confounding from external phenolic sources. This strategy allowed participants to follow their normal mixed diet while ensuring that the EVOO provided was the primary source of dietary phenolics during the intervention. The intervention lasted 4 weeks, and the healthy control group consumed EVOO under the same protocol as patients, with allocation to the two EVOO types made in the same proportions as in the patient groups.

**Figure 2:**
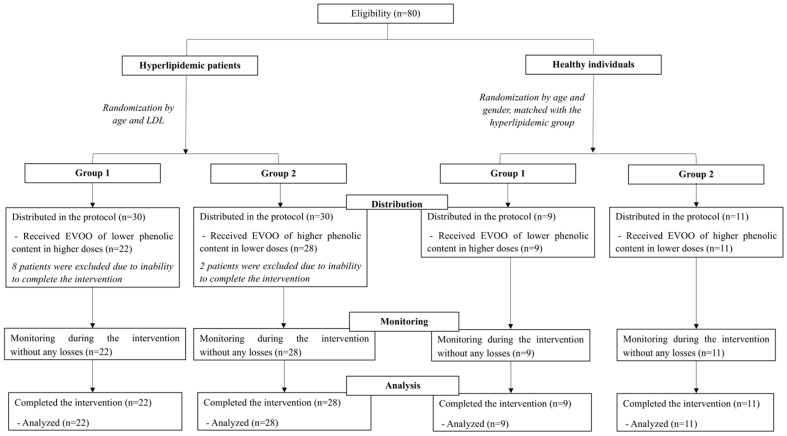
Flowchart of the study design [16].

The study enrolled 70 participants in total, comprising 50 patients with hyperlipidemia and 20 healthy controls who were matched for age and gender (Figure 2). Patients were randomized into two intervention groups according to the phenolic content of the EVOO provided, while controls were divided in the same manner. As a result, 22 patients were allocated to Group 1 (lower-phenolic EVOO of higher dose) and 28 patients to Group 2 (higher-phenolic EVOO of lower dose), with this distribution shaped by participant availability within strata and by attrition from individuals unable to complete the study protocol [16]. Compliance among those who remained in the trial was complete in both groups.

Both EVOO samples were produced in the Messinia region of Greece from the *Koroneiki* cultivar, harvested during the same season and processed in the same mill under identical cold-extraction, two-phase conditions to ensure comparable compositional characteristics. This controlled production approach minimized variability in fatty acid composition and matrix structure, allowing phenolic content to be the primary distinguishing factor between oils. Both samples met extra-virgin quality standards (Figure A3 and Figure A4) and were packaged in coded, identical dark glass bottles to maintain single-blind conditions. Oils were stored at 15–18 °C, protected from light, and used within three months of bottling to preserve their phenolic load. Adherence was monitored through weekly calls and container returns. Participants remained blinded to phenolic content and the rationale for phenolic dose selection, while investigators responsible for allocation and compliance were unblinded for accuracy.

The two EVOO samples were pre-selected from a larger batch based on their phenolic content, quantified using a validated quantitative proton nuclear magnetic resonance (^1^H-NMR) method [30]. This technique allows direct identification and quantification of individual phenolic constituents, including hydroxytyrosol and tyrosol derivatives, oleocanthal, oleacein, oleuropein aglycone, and other secoiridoids, without reliance on a single reference standard. The lower-phenolic oil contained 414 mg/kg total phenolics, whereas the higher-phenolic oil contained 1021 mg/kg. These values represent the sum of all quantified phenolics per kilogram of oil. Full compositional characterization confirmed that the two oils shared similar qualitative phenolic patterns and comparable tyrosol-to-hydroxytyrosol derivative ratios, differing primarily in the overall abundance of these compounds. This was essential for the study design, as biological effects may arise from individual phenolics or from synergistic interactions within the phenolic matrix rather than from total concentration alone.

### 2.3. Vascular Endothelial Function Assessment and Endpoints

Before the start and after the completion of the intervention, tissue oxygen saturation (StO_2_) was assessed using the NIRS method in combination with VOT. The operating principle of NIRS is based on the ability of the infrared light to reach skeletal muscle tissue, and to be absorbed by chromophore molecules, such as hemoglobin [31]. The Inspectra StO_2_ Tissue Oxygenation Monitor Model 650 (Hutchinson Technology, Hutchinson, MN, USA) was used for the measurement. All patients were measured at rest in a sitting position for 15 min. An opto diode was placed on the thenar muscle of the patients that recorded its tissue saturation. To induce ischemia through vascular occlusion of the brachial artery and brachial vein, the sphygmomanometer cuff was placed on the patient’s arm and the air inflation was set at 50 mmHg above the resting systolic pressure for 5 min, according to the literature [31]. Immediately after 5 min, the cuff was immediately released, resulting in the initiation of blood flow to the limb via the brachial artery (reperfusion phase) [31,32,33].

The basic parameters that were analyzed were: i. the average resting tissue oxygen saturation for 2 min before the start of VOT [baseline tissue oxygen saturation, StO_2_ (%)], ii. the tissue oxygen consumption rate in the ischemia phase [oxygen consumption rate, OCR (%/min)] with a starting value of ≤2% (0.98 times) of the average baseline StO_2_ (slope of decrease in the curve), iii. the reperfusion rate, which is an indicator of endothelial function [reperfusion rate, RR (%/s)] with a starting value of ≥5% (1.05 times) from the mean of baseline StO_2_ (slope of increase in the curve), iv. the time from maximum ischemia to the maximum tissue oxygen saturation recorded in the hyperemia phase [time from lowest to highest tissue oxygen saturation at the end of the occlusion period/time to hyperemia, TH (s)], and v. the recovery time of StO_2_ in the resting phase after the release of the cuff and the induction of reactive hyperemia [reactive hyperemia time, RHT (s)] which is an indicator of the vascular reserve of the microcirculation [31,32].

NIRS, especially when combined with the VOT, has been studied for reliability and reproducibility in vascular endothelial function and microcirculation assessment. Gerovasili et al. [32] showed that NIRS with VOT produces consistent measures across operators, with intra-class correlation coefficients (ICC) >0.80 for key parameters. In addition, Iannetta et al. [31] reported that NIRS-derived indices such as OCR and RR have good test–retest reliability, with coefficients of variation (CVs) generally <10–15% across repeated ischemic periods.

The primary endpoints included vascular endothelial function indices at baseline, during occlusion and during recovery, including StO_2_, OCR, RR, RHT and TH. Secondary endpoints included hemodynamic parameters such as systolic (SBP) and diastolic (DBP) blood pressure, oxygen saturation (SpO_2_) and heart rate (HR).

### 2.4. Statistical Analysis

Statistical analyses for the present post hoc study were conducted using data derived from our previously published randomized clinical trial, which primarily investigated the effects of high-phenolic extra virgin olive oil on the lipid profile of patients with hyperlipidemia [16]. This follow-up analysis was focused on vascular endothelial function and hemodynamic parameters as new primary and secondary endpoints, respectively.

The normality of data distributions was evaluated using the Kolmogorov–Smirnov test for samples ≥50 participants, complemented by visual inspection. Normality assumptions were met in all cases. Categorical variables were summarized as frequencies or percentages, and continuous variables as mean ± standard deviation. Group differences in means were assessed with independent-samples t-tests, while proportions were compared using chi-square tests.

Although only two measurements per participant (baseline and post-intervention) were available, linear mixed-effects models (LMMs) were applied due to their ability to handle within-subject correlations. Fixed effects included time (pre vs. post), group (patients vs. controls or EVOO group 1 vs. group 2), and the time × group interaction to determine whether temporal changes differed between groups. A random intercept for each subject accounted for inter-individual variability. The general model structure was:

Yij = β0 + β1(Timej) + β2(Groupi) + β3(Timej × Groupi) + ui + εij.

This approach is suitable for datasets with two repeated measures and is frequently used in clinical and nutritional research to test interaction effects, while also accommodating unbalanced data from dropouts more effectively than repeated-measures ANOVA.

Post hoc comparisons within and between groups were performed with Bonferroni correction to control for multiple testing and reduce type I error. Model fit was assessed by comparing the Akaike Information Criterion (AIC) values of models with and without interaction terms, which showed negligible differences (<2), supporting parsimony. Exploratory LMMs including a three-way interaction (time × group × gender) were fitted to evaluate gender-specific responses. All statistical analyses were performed using R software (version 4.3.2), employing the lme4 package for mixed-effects modeling and the emmeans package for post hoc comparisons. A two-tailed significance level of *p* < 0.05 was considered statistically significant.

## 3. Results

### 3.1. Baseline Characteristics

At baseline, patients and controls showed no significant demographic or hemodynamic differences (Table 1). The mean age was 52.2 ± 9.3 years for the patient group and 48.9 ± 8.7 years for the controls (*p* = 0.432). Gender distribution was balanced, with 24 men and 26 women among patients compared to 8 men and 12 women in the control group. Mean BMI was also comparable between groups (27.2 ± 4.2 kg/m^2^ in patients vs. 26.7 ± 5.1 kg/m^2^ in controls, *p* = 0.865). However, as expected, healthy individuals demonstrated better baseline vascular endothelial function compared to hyperlipidemic patients, as indicated by higher OCR (9.3 ± 1.2%/min vs. 7.1 ± 1.7%/min, respectively; *p* < 0.001), RR (4.4 ± 1.0%/s vs. 3.4 ± 1.1%/s, respectively; *p* = 0.001) and area of ischemia (117.8 ± 17.6 units × min vs. 96.8 ± 25.3 units × min, respectively; *p* = 0.002), as well as lower RHT (145.1 ± 24.6 s vs. 170.9 ± 28.3 s, respectively; *p* < 0.001).

### 3.2. Comparison Between Hyperlipidemic Patients and Healthy Individuals

**Figure 3:**
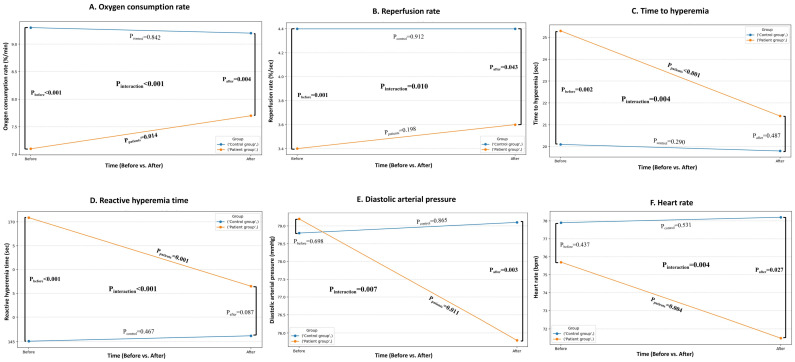
Comparison between hyperlipidemic patients and healthy individuals in variables with statistically significant interaction of time and group including (**A**) oxygen consumption rate, (**B**) reperfusion rate, (**C**) time to hyperemia, (**D**) reactive hyperemia time, (**E**) diastolic arterial blood pressure, and (**F**) heart rate.

**Figure A1:**
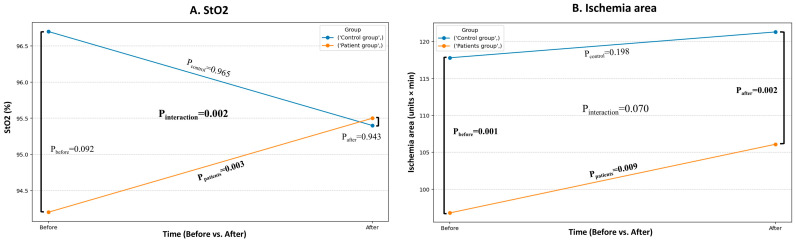
Comparison between hyperlipidemic patients and healthy individuals in (**A**) StO2 (*p_int_* = 0.002), (**B**) Ischemia area (*p_int_* = 0.070).

**Figure A2:**
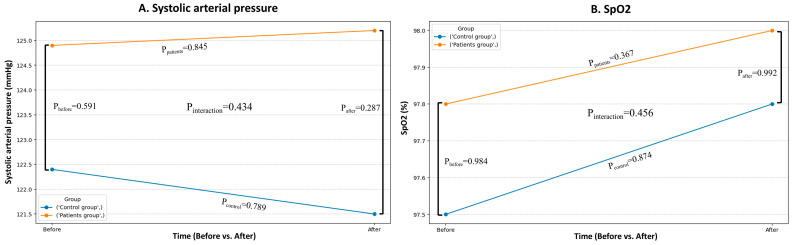
Comparison between hyperlipidemic patients and healthy individuals in (**A**) systolic arterial blood pressure (*p_int_* = 0.434), and (**B**) blood oxygen saturation (*p_int_* = 0.456).

**Figure A3:**
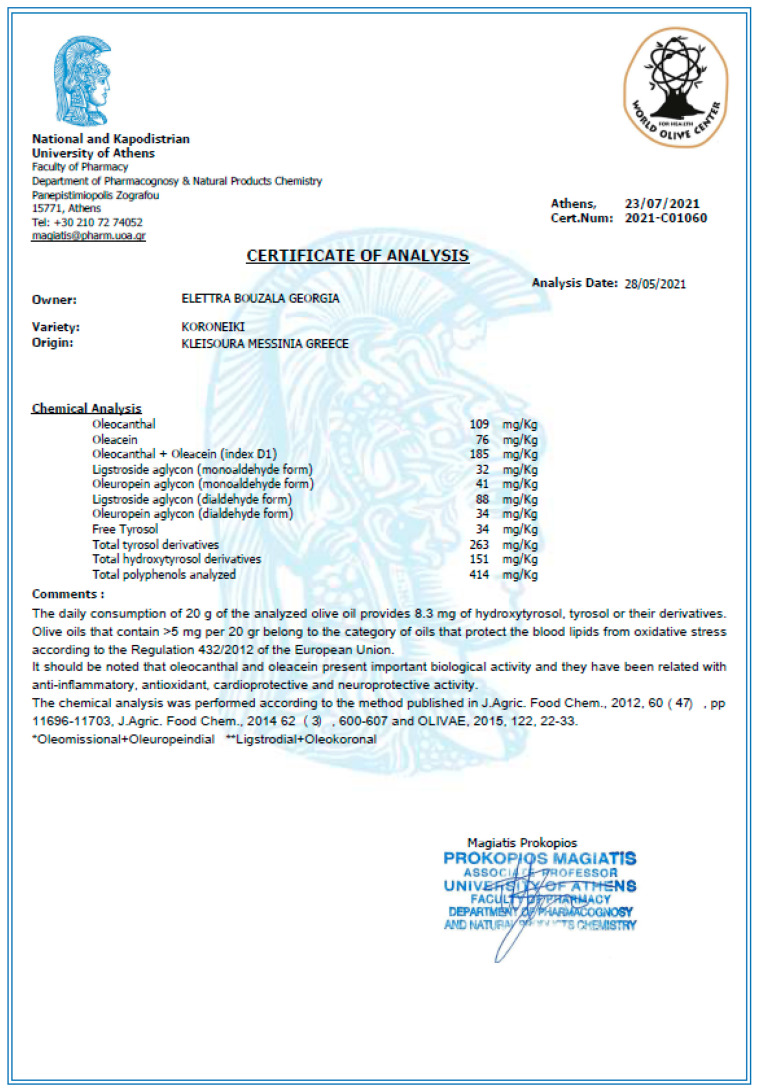
Certificate of analysis of EVOO of lower-phenolic content [63,64].

**Figure A4:**
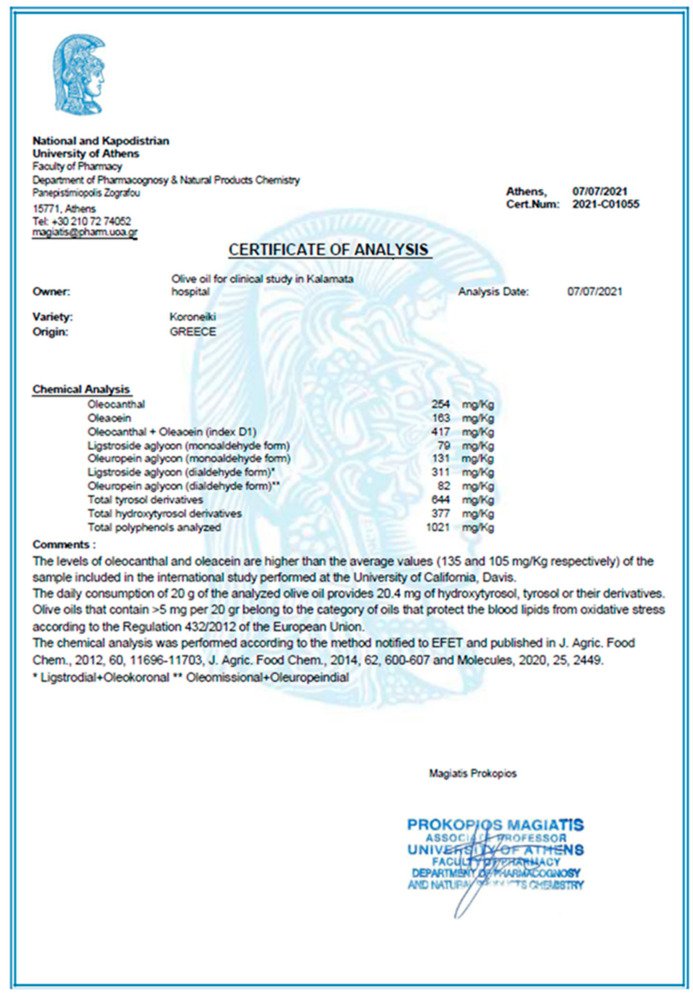
Certificate of analysis of EVOO of higher-phenolic content [30,63,64].

After the intervention, hyperlipidemic patients showed greater improvement in specific hemodynamic parameters and vascular endothelial function indices compared to healthy individuals. Specifically, in vascular endothelial function, a statistically significant interaction between time and group was obtained for StO_2_ (*p_int_* = 0.002; Figure A1), OCR (*p_int_* < 0.001; Figure 3A) and RR (*p_int_* = 0.010; Figure 3B) indicating a significant increase in patients compared to the non-significant change or decrease in healthy individuals. Moreover, a greater decrease in TH (*p_int_* = 0.004; Figure 3C) and RHT (*p_int_* < 0.001; Figure 3D) in hyperlipidemic patients compared to healthy individuals, was suggested. There were no statistically significant interactions as regards the remaining vascular endothelial function indices (Figure A1). In hemodynamic parameters, diastolic arterial blood pressure (*p* = 0.007; Figure 3E) and heart rate (*p* = 0.004; Figure 3F) significantly decreased in hyperlipidemic patients compared to healthy individuals. The rest of the parameters showed similar change between the 2 groups (Figure A2).

### 3.3. Comparison Between the 2 Groups of Hyperlipidemic Patients of Different EVOOs

The comparison of baseline characteristics between the two groups of hyperlipidemic patients did not reveal statistically significant differences in demographics, hemodynamic parameters and vascular endothelial function indices, making the groups suitable for comparison (Table 2).

In the endothelial function, RR significantly increased in Group 2 compared to Group 1 (*p* = 0.049; Table 3). Moreover, TH showed a trend for further improvement in Group 2, but did not reach statistical significance (*p* = 0.096; Table 3).

In the hemodynamic parameters, SAP was significantly lower in Group 2 compared to Group 1 after the intervention (*p* = 0.048; Table 4).

### 3.4. The Effect of Gender on the Intervention in Patients with Hyperlipidemia

**Figure 4:**
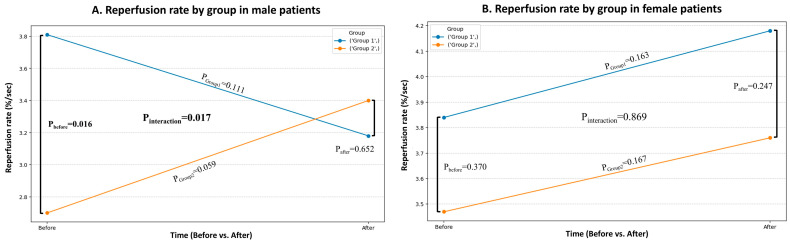
Comparison between the 2 patient groups in reperfusion rate with statistically significant interaction of time and group in (**A**) male patients, but not in (**B**) female patients.

Regarding the potential impact of gender on vascular endothelial function and hemodynamic responses to EVOO, significant gender-related differences were revealed (Table A1). Specifically, reperfusion rate showed a significant interaction among gender, group and time (*p* = 0.025), suggesting that the impact of the intervention over time is different for males (Figure 4A) and females (Figure 4B). A decrease in RR was observed in males of Group 1 after the intervention (*p* = 0.111) and an increase in males of Group 2 (*p* = 0.059), indicating a significant time × group × gender interaction. In contrast, female patients in both groups showed similar trends toward increased RR, but without a significant interaction effect.

## 4. Discussion

The present clinical study showed that the consumption of EVOO had beneficial effects on the endothelial function, leading to an increase in resting tissue saturation, oxygen consumption rate and reperfusion rate, and a decrease in the times of maximum hyperemia and hyperemia recovery, indicating modest but statistically significant improvements in microcirculation after the intervention. Hemodynamic parameters also showed small but measurable changes, including a decrease in diastolic blood pressure and heart rate. Another important finding of the study is that the higher-phenolic EVOO in lower dosages had significant benefits in further increasing the reperfusion rate, and reducing systolic blood pressure, compared to the lower-phenolic EVOO in higher dosages, suggesting a potential improvement of vascular endothelial function and hemodynamic status, although the magnitude of change was modest. Finally, it was observed that gender played an important role in a specific indicator of vascular function, the reperfusion rate, with males in the 2 groups showing different changes in this parameter, suggesting that the intervention may have a differentiated effect depending on gender, possibly due to different responses to treatment. However, these improvements must be interpreted cautiously, as several unmeasured factors such as dietary variability, physical activity, medication adherence, and baseline differences in microvascular function could have influenced the observed responses.

In our previous work [16], EVOO consumption was demonstrated to produce favorable changes in the lipid profile of hyperlipidemic patients, including significant increases in HDL cholesterol and reductions in lipoprotein(a). These improvements in lipoprotein quality and composition are clinically relevant, as dysfunctional HDL and oxidized LDL are strongly implicated in vascular injury and endothelial dysfunction [23,34,35]. In the present study, these observations were extended by showing that EVOO also exerts direct benefits on vascular endothelial function. The enhancement of endothelial reactivity observed here is consistent with the lipid-modifying effects reported earlier, since improved lipid metabolism reduces oxidative stress and inflammatory burden at the vascular interface [36,37]. These findings may suggest a complementary mechanism by which EVOO not only optimizes lipid parameters but also promotes endothelial health, thereby contributing to a more comprehensive cardioprotective effect. They also highlight the importance of EVOO as part of a nutritional strategy for cardiovascular protection and indicate its possible role as a supportive non-pharmacological strategy in the management of hyperlipidemia and endothelial dysfunction. The improvements observed in both blood lipid profile and vascular endothelial function, as well as the evaluation of hemodynamic parameters, provide a new perspective in the formulation of targeted nutritional strategies and add new evidence to the literature, providing preliminary evidence that supports a link between nutritional interventions and cardiovascular risk modulation. It is also important to consider that some of the observed changes may reflect natural intra-individual variability rather than a pure effect of the intervention, particularly given the brief duration of the study.

This study is one of the first human interventions to integrate NIRS with the VOT protocol for the assessment of microvascular function in response to high-phenolic EVOO. This combined methodology offers new insights into early endothelial and microcirculatory adaptations that are not captured by traditional conduit-artery measurements. A previous study by Cutruzzolà et al. [38] showed that consumption of olive oil enriched with monounsaturated fats (MUFA) and polyphenols increases blood flow and improves endothelial function in patients with type 1 diabetes, compared to other saturated fats such as butter, which worsen vascular endothelial dysfunction. Using the endothelium-dependent vasodilation method, an increase in vasodilation was observed (*p* = 0.007), compared to a group of patients who consumed butter. The study by Njike et al. [39] focused on the direct effects of polyphenol-rich olive oil on vascular endothelial function in patients at risk for developing type 2 diabetes mellitus. After consuming 50 mL of high-phenolic olive oil, a significant improvement in endothelial function was observed compared to consumption of processed low-phenolic olive oil (vasodilation at FMD 1.2 ± 6.5% vs. −3.6 ± 3.8%, respectively; *p* = 0.009). Similarly, our study recorded improvements in vascular microcirculation and tissue oxygen consumption (OCR), confirming the positive effect of EVOO. In fact, the group consuming olive oil with a higher-phenolic content showed a statistically significant improvement in reperfusion rate, an indicator of endothelial function [32], compared to the group consuming olive oil with a lower-phenolic content. Important methodological similarities and differences emerge when comparing our findings with the existing literature. Cutruzzolà et al. reported a significant improvement in FMD in patients with type 1 diabetes after consumption of MUFA- and polyphenol-enriched olive oil [38], whereas Njike et al. demonstrated acute FMD enhancement following intake of high-phenolic olive oil in individuals at risk for type 2 diabetes [39]. Both studies assessed vascular function at the conduit-artery level using ultrasound-based FMD, in contrast to the present study, which evaluated microvascular endothelial responses through NIRS-derived indices. Despite these methodological differences, all three studies suggest that phenolic-rich EVOO can beneficially modulate vascular reactivity, with our work extending this concept to the microcirculatory level in hyperlipidemic patients. However, variations in vascular assessment techniques, participant characteristics, and phenolic dosing likely contribute to heterogeneity in the magnitude of reported effects and limit the extent to which direct comparisons can be made.

In a less recent study, Ruano et al. [40] compared high (400 ppm) vs. low (80 ppm) phenolic virgin olive oil in hyper-cholesterolemic adults and found improved ischemic reactive hyperemia 2–4 h after the meal, correlating with increases in nitrates/nitrites (NO(x)) and decreases in lipid peroxidation markers. A broader perspective from Woodward et al.’s [41] review of microvascular endpoints highlights how polyphenol source, dose, duration, and assessment technique critically determine the size and consistency of vascular responses. These studies reinforce our findings but also underscore why effect sizes in our short-term microvascular NIRS intervention were modest compared with large conduit-artery FMD studies.

In addition, significant differences in the response between men and women on this index were shown in the present study, suggesting possible physiological differences in endothelial response to polyphenols. This variation may be related to hormonal and physiological differences between the sexes that influence the response to polyphenols. However, the number of participants per gender subgroup was small, and therefore this observation should be considered exploratory and not interpreted as a definitive gender-related effect.

The use of NIRS as the main methodology for assessing microcirculation offered unique analytical capabilities, providing detailed data on dynamic changes in the vascular endothelium and their connections to the improvement of the lipidemic profile. It thus provides a comprehensive picture of vascular endothelial function, which is an added value to the literature. In contrast to traditional methods, such as measuring endothelium-dependent vasodilation (FMD) of the brachial artery, which has high variability due to different experience between sonographers or even between measurements by the same sonographer [42,43], the NIRS method provides more accurate and repeatable measurements without high variability, ideal for evaluating interventions aimed at improving vascular health [31].

Beyond acute FMD studies, several randomized trials have examined the impact of phenolic-rich olive oil on microvascular or hemodynamic endpoints. Moreno-Luna et al. [44] reported that a polyphenol-rich olive oil diet, compared with polyphenol-free olive oil, reduced systolic and diastolic blood pressure by approximately 8 and 7 mmHg, respectively, and improved ischemia-induced hyperemia in young women with mild hypertension. Valls et al. [45] showed that a single dose of functional virgin olive oil enriched with its own phenolic compounds enhanced ischemic reactive hyperemia and reduced post-prandial oxidized LDL and inflammatory markers in hypertensive patients [Ref]. In healthy adults, Sarapis et al. [46] observed modest but significant reductions in peripheral and central systolic blood pressure after three weeks of high-polyphenol EVOO compared with low-polyphenol olive oil. Taken together, these trials complement our findings by indicating that phenolic-rich EVOO exerts generally modest improvements in endothelial or hemodynamic parameters across different risk groups, with the magnitude of change influenced by baseline risk, phenolic dose, and intervention duration.

Hyperlipidemia is closely linked to vascular inflammation, primarily through excessive generation of ROS, which diminish NO synthesis and impair vasodilation [22,47,48]. This oxidative stress promotes monocyte activation, upregulation of adhesion molecules such as ICAM-1, and enhances thrombogenic activity [49,50]. Lipid fractions play a direct role in this process: elevated LDL and apolipoprotein B (ApoB) levels drive lipid deposition in the arterial wall, triggering oxidative and inflammatory injury to the endothelium [23,51,52]. Conversely, HDL and apolipoprotein A1 (ApoA1) exert protective actions by facilitating cholesterol efflux from macrophages and augmenting endothelial NO availability [53,54]. The imbalance between atherogenic and anti-atherogenic lipids therefore destabilizes endothelial homeostasis and elevates cardiovascular risk. Polyphenols in EVOO, particularly hydroxytyrosol, oleacein, and oleuropein aglycone, exhibit potent antioxidant and anti-inflammatory properties that directly support endothelial health. By neutralizing ROS, these compounds preserve NO bioavailability and improve vascular relaxation [55,56]. From a mechanistic standpoint, the phenolic profile of the oils used in this trial is consistent with the current understanding of structure–activity relationships in EVOO phenolics. Hydroxytyrosol and oleuropein aglycone, which contain ortho-dihydroxyl groups on the aromatic ring, exhibit strong radical-scavenging capacity and protect against oxidative endothelial injury, while also downregulating adhesion molecules such as VCAM-1 and ICAM-1 and inflammatory cytokines in endothelial cells [25,57,58]. Secoiridoids such as oleocanthal and oleacein possess dialdehydic structures that confer potent anti-inflammatory activity, including inhibition of COX-1/COX-2, iNOS and NF-κB–related pathways, which are critically involved in vascular inflammation and atherogenesis [57,59,60]. These structural features are thought to underlie the ability of EVOO phenolics to preserve nitric oxide bioavailability, limit oxidative modification of LDL, and attenuate endothelial activation [61,62]. In our study, the higher-phenolic EVOO, enriched in these secoiridoids and hydroxytyrosol derivatives, was associated with modest but favorable changes in microvascular reperfusion and blood pressure, which is consistent with the notion that both the amount and structural characteristics of EVOO phenolics contribute to vascular protection.

The superior effects of the higher-phenolic EVOO given at a lower dose, previously attributed to an enhanced polyphenol-to-lipid ratio facilitating intestinal absorption [16], appear to extend beyond lipid regulation to vascular function. In the present study, this formulation was associated with improvements in lipid parameters and a reduction in systolic blood pressure; however, these changes were modest and should be interpreted with caution, given the limited sample size. These vascular benefits are consistent with the hypothesis that improved bioavailability of phenolic compounds augments their antioxidant and vasodilatory actions, thereby complementing lipid-lowering effects and providing a broader cardioprotective profile compared to the lower-phenolic EVOO, even if it is consumed at higher doses. Although several reports attribute the vascular benefits of EVOO to antioxidant and anti-inflammatory pathways, the degree to which these mechanisms contribute varies across studies. This variability may reflect differences in analytical methods used to quantify phenolics, the relative abundance of specific compounds such as oleocanthal or oleacein, and the timing of vascular measurements. Such discrepancies underscore the need for more standardized methodological approaches.

A major strength of this study is its randomized design, which minimized allocation bias and ensured balanced distribution across intervention groups. The use of two EVOOs with different phenolic concentrations but standardized total polyphenol intake, allowed for direct comparison of polyphenol concentration versus oil volume, an innovative feature not commonly addressed in nutritional trials. Participant adherence was excellent, supported by weekly monitoring and objective container checks. Additionally, the inclusion of both hyperlipidemic patients and healthy individuals broadens the relevance and clinical applicability of the findings. Finally, the use of NIRS, a non-interventional and reliable method for the assessment of vascular endothelial function, helps us to better understand pathophysiology between blood lipid profile and vascular health.

Limitations have been previously described in detail [16]. In summary, recruitment was based on voluntary participation from a specific geographical area, which may introduce selection bias and limit generalizability. The absence of a true placebo group prevents firm conclusions about whether the observed effects were due solely to polyphenols or also to EVOO’s fatty acid content. The short 4-week duration restricted assessment of long-term outcomes and durability after discontinuation. Moreover, small sample size, particularly for gender-based comparisons, may have limited statistical power. Beyond the small sample size, the recruitment of patients from a single lipid clinic may introduce selection bias, potentially limiting generalizability. Although several outcomes reached statistical significance, the effect sizes were modest, and the short 4-week intervention limits the strength of any mechanistic or clinical interpretations. Finally, although participants were instructed to avoid external sources of polyphenols, residual dietary intake variation cannot be fully excluded as a confounder. Taken together, while the findings are encouraging, the influence of potential confounders and the exploratory nature of the study design underscore the need for larger, rigorously controlled trials before drawing firm conclusions.

Overall, while the referenced literature consistently supports a beneficial role of EVOO in vascular health, the diversity in study protocols, phenolic profiles, and measurement techniques indicates that findings across studies should be compared with caution. Our results align with this body of evidence but represent a modest effect within the context of a short, lower-powered intervention.

## 5. Conclusions

In conclusion, short-term consumption of high-phenolic EVOO led to modest but statistically significant improvements in vascular endothelial function of patients with hyperlipidemia, including an increase in reperfusion rate of approximately 12–15%, an improvement in oxygen consumption rate of 8–10%, and small reductions in diastolic blood pressure of 2–3 mmHg. The use of a higher-phenolic EVOO at a lower dose appeared to be decisive for further improving systolic arterial blood pressure and RR among hyperlipidemic patients, compared to a lower-phenolic EVOO at a higher dose. These findings suggest that not only the total polyphenol intake but also the polyphenol-to-lipid ratio is critical in determining vascular and metabolic benefits. Moreover, gender was shown to have a significant impact on vascular endothelial function, with higher-phenolic EVOO demonstrating a better improvement among males compared to the similar improvement among females. While these findings are encouraging, they are limited by the small sample size and short intervention duration and should therefore be interpreted as exploratory. Larger and longer-term trials are required to confirm these early physiological effects and clarify their potential clinical implications.

## Figures and Tables

**Table 1:** Baseline demographic characteristics and endothelial function markers and hemodynamic parameters of 70 hyperlipidemic patients and healthy individuals.

Demographics	Hyperlipidemic Patients	Healthy Individuals	*p* Value *
Number of patients (*N*)	50	20	
Gender (Males/Females)	24/26	9/11	0.954
Age (years)	52.2 ± 9.3	48.9 ± 8.7	0.432
BMI (kg/m^2^)	27.2 ± 4.2	26.7 ± 5.1	0.865
**Hemodynamic parameters**			
Systolic arterial pressure (mmHg)	124.9 ± 12.5	122.4 ± 13.1	0.311
Diastolic arterial pressure (mmHg)	79.2 ± 8.1	78.8 ± 7.9	0.698
Blood oxygen saturation (%)	97.8 ± 1.1	97.5 ± 1.3	0.951
Heart rate (bpm)	75.7 ± 13.5	77.9 ± 12.4	0.437
**Vascular endothelial function indices**			
StO_2_ (%)	94.2 ± 2.1	96.7 ± 1.8	0.092
OCR (%/min)	7.1 ± 1.7	9.3 ± 1.2	**<0.001**
RR (%/s)	3.4 ± 1.1	4.4 ± 1.0	**0.001**
TiH (s)	25.3 ± 7.4	20.1 ± 5.6	0.006
RHT (s)	170.9 ± 28.3	145.1 ± 24.6	**<0.001**
Ischemia area (units × min)	96.8 ± 25.3	117.8 ± 17.6	**0.002**

* Comparison of baseline values with *t*-test for independent samples and comparison of percentages with χ^2^ test. Abbreviations: BMI, body mass index; StO_2_, resting tissue oxygen; OCR, oxygen consumption rate; RR, reperfusion rate; TiH, time to hyperemia; RHT, reactive hyperemia time. Values in bold indicate a statistically significant findings, taking into account the Bonferroni correction for quantitative indicators (*p* ≤ 0.005 = 0.05/10).

**Table 2:** Baseline demographic characteristics between the 2 groups of patients with hyperlipidemia who participated in the study.

Demographics	Group 1	Group 2	*p* Value *
Number of patients (*N*)	22	28	0.433
Gender (Males/Females)	11/11	13/15	0.802
Age (years)	52.7 ± 8.0	51.8 ± 10.3	0.736
BMI (kg/m^2^)	26.5 ± 3.9	27.8 ± 4.5	0.289
**Hemodynamic parameters**			
Systolic arterial pressure (mmHg)	124.1 ± 12.2	125.5 ± 13.0	0.697
Diastolic arterial pressure (mmHg)	79.6 ± 7.9	79.0 ± 8.3	0.815
Blood oxygen saturation (%)	98.0 ± 1.2	97.8 ± 1.1	0.529
Heart rate (bpm)	75.1 ± 13.1	76.3 ± 14.1	0.751
**Vascular endothelial function indices**			
StO_2_ (%)	93.9 ± 2.0	94.4 ± 2.3	0.292
OCR (%/min)	7.6 ± 1.7	6.7 ± 1.6	0.090
RR (%/s)	3.8 ± 1.1	3.1 ± 1.0	0.021
TiH (s)	23.6 ± 5.2	26.6 ± 8.5	0.288
RHT (s)	171.9 ± 25.8	170.0 ± 30.6	0.827
Ischemia area (units × min)	104.8 ± 24.7	90.6 ± 24.3	0.018

Group 1, patients who received EVOO of lower-phenolic content at a higher dose. Group 2, patients who received EVOO of higher-phenolic content at a lower dose. * Comparison of baseline values with t-test for independent samples and comparison of percentages with χ^2^ test. Abbreviations: BMI, body mass index; StO_2_, resting tissue oxygen; OCR, oxygen consumption rate; RR, reperfusion rate; TiH, time to hyperemia; RHT, reactive hyperemia time. Statistical significance after Bonferroni correction for quantitative indicators is *p* ≤ 0.005 (=0.05/10).

**Table 3:** Beta coefficients (β) derived from linear mixed models to evaluate the interaction of time × group on endothelial function.

Values	Estimator (β)	95% CI	*p*-Value
**Resting tissue oxygen (%)**			
(Intercept)	95.09	94.11 to 96.07	**<0.001**
Time (After)	1.18	−0.44 to 2.18	0.071
Group (Group 2)	0.77	−0.54 to 2.08	0.254
Time × Group (After × Group 2)	0.28	−1.96 to 1.40	0.743
**Oxygen consumption rate (%/min)**			
(Intercept)	−7.56	−8.22 to −6.90	**<0.001**
Time (After)	−0.63	−1.36 to 0.10	0.098
Group (Group 2)	0.81	−0.06 to 1.69	0.072
Time × Group (After × Group 2)	0.001	−0.97 to 0.98	0.995
**Reperfusion rate (%/s)**			
(Intercept)	3.83	3.39 to 4.27	**<0.001**
Time (After)	−0.14	−0.60 to 0.31	0.539
Group (Group 2)	−0.71	−1.30 to −0.12	**0.021**
Time × Group (After × Group 2)	0.62	0.02 to 1.23	**0.049**
**Time to maximum hyperemia (s)**			
(Intercept)	23.55	20.83 to 26.26	**<0.001**
Time (After)	−2.09	−4.88 to 0.70	0.148
Group (Group 2)	3.06	−0.57 to 6.69	0.102
Time × Group (After × Group 2)	−3.23	−6.96 to 0.49	0.095
**Reactive hyperemia time (s)**			
(Intercept)	171.86	160.30 to 183.43	**<0.001**
Time (After)	−15.55	−28.08 to −3.01	**0.018**
Group (Group 2)	−1.79	−17.25 to 13.66	0.820
Time × Group (After × Group 2)	2.15	−14.60 to 18.90	0.802
**Ischemia area (units × min)**			
(Intercept)	−104.83	−114.26 to −95.34	**<0.001**
Time (After)	−8.65	−18.84 to 1.53	0.102
Group (Group 2)	14.22	1.58 to 26.86	**0.030**
Time × Group (After × Group 2)	−1.02	−14.63 to 12.59	0.883

Reference categories were ‘Before’ for time and ‘Group 1’ (lower-phenolic EVOO, higher dose) for group; thus, coefficients for Time (After), Group (Group 2), and their interaction are interpreted relative to these baselines. Bold values indicate statistical significance.

**Table 4:** Beta coefficients (β) derived from linear mixed models to evaluate the interaction of time × group on hemodynamic parameters.

Values	Estimator (β)	95% CI	*p*-Value
**Systolic arterial pressure (mmHg)**			
(Intercept)	124.09	117.96 to 130.23	**<0.001**
Time (After)	3.68	−0.70 to 8.06	0.106
Group (Group 2)	1.41	−6.79 to 9.61	0.737
Time × Group (After × Group 2)	−6.04	−11.89 to −0.19	**0.048**
**Diastolic arterial pressure (mmHg)**			
(Intercept)	79.55	75.48 to 83.61	**<0.001**
Time (After)	−2.64	−6.52 to 1.25	0.190
Group (Group 2)	−0.55	−5.97 to 4.88	0.844
Time × Group (After × Group 2)	−1.47	−6.66 to 3.72	0.581
**Heart rate (beats per min)**			
(Intercept)	75.05	69.76 to 80.33	**<0.001**
Time (After)	−3.23	−7.38 to 0.92	0.134
Group (Group 2)	1.24	−5.82 to 8.30	0.732
Time × Group (After × Group 2)	−1.74	−7.28 to 3.81	0.542
**Blood oxygen saturation (%)**			
(Intercept)	97.95	97.49 to 98.42	**<0.001**
Time (After)	0.14	−0.32 to 0.60	0.563
Group (Group 2)	−0.20	−0.83 to 0.42	0.524
Time × Group (After × Group 2)	0.01	−0.61 to 0.62	0.984

Reference categories were ‘Before’ for time and ‘Group 1’ (lower-phenolic EVOO, higher dose) for group; thus, coefficients for Time (After), Group (Group 2), and their interaction are interpreted relative to these baselines. Bold values indicate statistical significance.

## Data Availability

The data presented in this study are available on request from the corresponding author due to privacy of personal information.

## Abbreviations

The following abbreviations are used in this manuscript:

EVOO	Extra virgin olive oil
NIRS	Near-infrared spectroscopy
VOT	Vascular occlusion technique
BMI	Body mass index
StO_2_	Resting tissue oxygen
OCR	Oxygen consumption rate
RR	Reperfusion rate
TiH	Time to hyperemia
RHT	Reactive hyperemia time
SBP	Systolic blood pressure
DBP	Diastolic blood pressure
SpO_2_	Oxygen saturation
HR	Heart rate

## Appendix A

**Table A1:** Differences in the vascular endothelial function and hemodynamic parameters based on the impact of gender.

Vascular Endothelial Function Indices	Time × Group × Gender Interaction (*p* Value)	Group × Gender Interaction (*p* Value)	Time × Gender Interaction (*p* Value)
Resting tissue oxygen (%)	0.696	0.725	0.550
Oxygen consumption rate (%/min)	0.209	0.706	0.910
Reperfusion rate (%/s)	**0.025**	0.914	0.342
Time to maximum hyperemia (s)	0.417	0.279	0.062
Reactive hyperemia time (s)	0.654	0.662	0.639
Ischemia area (units × min)	0.483	0.615	0.158
**Hemodynamic parameters**			
Systolic arterial pressure (mmHg)	0.621	0.689	0.545
Diastolic arterial pressure (mmHg)	0.281	0.315	0.244
Heart rate (beats per min)	0.543	0.673	0.309
Blood oxygen saturation (%)	0.187	0.214	0.301

The analysis is performed using linear mixed models to determine the existence of statistical significance in the interaction of time, gender, and group on each parameter of interest. Bold values indicate statistical significance.
